# *I simply have to accompany my parents to sell*,* nothing else!*: a multi-method exploration of barriers and facilitators of extracurricular physical activity among Mexican schoolchildren

**DOI:** 10.1186/s12966-025-01716-9

**Published:** 2025-03-03

**Authors:** Gabriela Argumedo, Michael W. Beets, Jesús Garcés, Fritz Culp, Edgar Denova, Rocío Alvarado-Casas, Anabelle Bonvecchio-Arenas, James F. Thrasher, Alejandra Jáuregui

**Affiliations:** 1 Centro de Investigación en Nutrición y Salud, Instituto Nacional de Salud Pública Ciudad de México, Ciudad de México, México; 2 Arnold School of Public Health, University of South Carolina, Columbia, USA

**Keywords:** Extracurricular activities, Physical activity, Children, Screen time, Qualitative study

## Abstract

**Background:**

The time spent physically active outside of school (e.g., extracurricular physical activity) is an important contributor to children’s total daily physical activity for health and well-being. Little is known about the opportunities available to children to engage in extracurricular physical activity from low- to middle-income countries. This study aims to answer the question: What are the main perceived barriers and facilitators of extracurricular physical activity among school-age children in Mexico?

**Methods:**

A multi-method cross-sectional study was performed. Six focus groups with children (aged 9–12 years), six focus groups with parents, 10 one-on-one interviews with parents, 12 interviews with teachers, and six interviews with head teachers were conducted across Campeche, Morelos, and Mexico State, Mexico. A questionnaire was applied to explore children’s physical activity frequency and preferences for time inside and outside of school. Qualitative data analyses were performed with inductive thematic analysis supported with NVivo software. Quantitative data were analysed with descriptive statistics using IBM SPSS 26.

**Results:**

Three main themes summarise the study’s findings: (1) how children spend their time outside of school, (2) the places that children access, and (3) the social environment for physical activity outside of the school. The data suggest that children in Mexico dedicate their spare time to screen, work, do housework, or perform unstructured physical activity mostly at home instead of playing sports or actively outdoors. Family support, enjoyment of physical activity, access to programs and facilities, time, living in a housing complex with open common areas, and mild weather were important facilitators identified. 69.4% of children engage in extracurricular physical activity, none of which was provided by schools. More children commute by walking than riding a bike to and from school. Children living inland spent three times more time at home compared to those in seafront areas.

**Conclusions:**

Children rely on their families to partake in extracurricular structured physical activity. Policies targeting children’s health and well-being should include school-based extracurricular physical activity programs.

**Supplementary Information:**

The online version contains supplementary material available at 10.1186/s12966-025-01716-9.

## Introduction

Regular physical activity contributes to health, well-being, academic outcomes, and the prevention and treatment of noncommunicable diseases [1, 2]. The World Health Organisation (WHO) recommends that children and adolescents accumulate an average of 60 min per day of moderate to vigorous physical activity (MVPA) [3]; however, few meet this standard regularly. Physical activity is a multidimensional behaviour [4] consisting of different domains, including leisure, occupational, active travel, domestic [5], as well as characteristics (e.g., type, intensity, duration), aspects related to the individual (e.g., attitudes, profession), time or timing (e.g., season, working hours), physical context (e.g., weather, location), and social context (e.g., friends, family) [6]. Physical activity is also organised into two broad contexts, structured and unstructured, where the former is planned and has objectives, and the latter refers to free-time or leisure play [7]. Children’s and adolescents’ extracurricular physical activity (physical activity outside of school) is an important contributor to meeting physical activity recommendations and contributes to their physical fitness and body composition [8, 9].

An important aspect of designing effective interventions to promote physical activity is understanding the contexts where children are most active and what factors influence their participation in physical activity. Home, school, and neighbourhood are the main settings where children are physically active [10, 11]. A study based on data from high-income countries (HICs) indicated that active transportation is also an important contributor to MVPA among children and adolescents [12]. As part of the social context, children and adolescents rely on family support to engage in some form of physical activity [13]. Family support could be tangible (e.g., enrolment fee coverage) or intangible (verbal encouragement concerning physical activity) [14]. However, most data concerning children’s physical activity are from studies from HICs [10, 15]. Children in low-to-middle-income countries (LMICs) may not have similar opportunities or access to the contexts in which children from HICs typically engage in physical activity [15]. In LMICs, work may also serve as a source of physical activity for children, particularly in contexts where they are involved in economic or household responsibilities [16]. The barriers and facilitators for accessing physical activity may be unique based on context and income level specific to LMICs and thus require differing intervention strategies.

In Mexico, approximately 70% of children do not meet the physical activity recommendations, whereas 82% exceed 2 h of screen time per day [17]. During the school day, children spend around 4.5 h at school. The only opportunities children have to participate in physical activity at school are physical education, which averages of 15–29 min of MVPA per session and is typically offered only once a week, along with daily recess, which provides an average of 15 min per day [18, 19]. Little is known about how children spend their time outside of school, the places they access, and how these places contribute to their physical activity. In addition, 19.2% have oveweight and 18.1% obesity [20]. Given the importance of extracurricular physical activity to children’s total daily physical activity and healthy weight, as well as the lack of data regarding children’s opportunities from LMICs to engage in physical activity outside the school day, this study aims to respond the question: What are the main perceived barriers and facilitators of extracurricular physical activity among school-age children in Mexico?

## Methods

### Design

This study is part of a larger multimethod study that included qualitative and quantitative data methods titled *Multidimensional intervention to improve healthy life and state of nutrition and health in Mexican schoolchildren: Development of an intervention platform*,* clinical trial*. *Randomized and proposed escalation.* The study protocol received ethical approval from the authors’ institutional ethics board (CI:1542, P19-18). The current study reports findings from qualitative data related to the study´s needs assessment phase focused on physical activity participation outside of school. The study uses an essentialist approach to gather participants’ experiences and semantic meanings [21]. The study design was cross-sectional, and data were collected via questionnaires, focus groups, and individual interviews among the school community, children, and parents in three states in Mexico. Data collection was conducted from January to July 2022, once the restrictions of the COVID-19 pandemic were lifted.

### Participants

The study was conducted in elementary public schools in Cuernavaca, Morelos; Ciudad del Carmen, Campeche; and Toluca, Mexico State. In the included states, < 5% of the children did not attend school, almost 50% lived in poverty [22] and differ in terms of environmental conditions, including seaside or urban density (Table 1). Using a pragmatic approach, two schools per state were randomly selected in collaboration with local education authorities, for a total of six schools. Eligible schools were public schools, located in urban areas with low socioeconomic backgrounds and had a population of at least 1,000 children with at least two groups per grade level and operated from 8:00–12:30 h. Based on these criteria, local education authorities selected two schools within each state for the research team to conduct research activities. The school staff was contacted by the local education administration, which assigned the school participants; then, the school staff assigned the school class to participate in the study. All 9–12-year-old 4th-grade school children were eligible. Parents, teachers, and head teachers provided informed consent, while children were informed about the study and asked to provide written passive assent and verbal confirmation to accept just before participating at any measure.

**Table 1:** Characteristics of the study participants´ context

	Characteristics	Region						
		South			Centre			
		State	Municipality		State	Municipality	State	Municipality
Environmental		Campeche	Carmen	Campeche	Morelos	Cuernavaca	Mexico State	Toluca
	% urban population [23]	75%	88.0%	90.0%	82%	94.3%	87%	90.0%
	% poverty^a^ [24]	45.1%			41.1%		42.9%	
	% of children engaged in inadequate household chores [25]	7.1%			6.5%		4.8%	
	Crime rate per 100,000 inhabitants [26]	27,727			32,333		36,583	
	Degree of marginalisation^b^ [27]	High	Very Low	Very Low	Medium	Very Low	Low	Very Low
	% children 6–11 years who do not attend to school [28]	4.9%	5.6%	2.3%	4.2%	3.6%	4.6%	4.3%
	Weather/environment [23]	Seafront Warm subhumid climate. Average annual temperature 26–7 °C.			Inland Warm subhumid weather. Average annual temperature 21.5 °C		Inland Subhumid climate. Average annual temperature is 14.7 °C	
Leisure programs								
	Parks for children	209	39	29	91	2	201	8
	Sports areas	317	38	34	239	6	1431	17
	Municipality/regional provision	Sports facilities, trained coaches and programs coordinated by the Institutional Sport Promotion Program [23].			Sports activities available for children at the local Sports Unit [24] Dancing options available at the Centro Morelense de las Artes [25] Free soccer classes coordinated by the Sports Institute [26]		Physical activity program coordinated by the Instituto Municipal de Cultura Física y Deporte de Toluca [27, 28]	

Note. ^a^= Having at least one social deprivation within six indicators (educational lag, access to health services, access to social security, quality and spaces of housing, basic services in housing, and access to food); this suggests family income is insufficient to acquire the goods and services required to meet both food and non-food needs

^b^= The Marginalisation Index is a multidimensional measure of the deprivations intensity, assesing nine forms of exclusion across four dimensions: education, housing, population distribution and monetary income. It categorizes geostatistical units based on the socioeconomic deficiencies their populations face, with higher marginalization levels indicating more severe deprivation

### Procedures

For the qualitative component, the sampling was designed to ensure data saturation and to provide voice to participants from diverse contexts and country regions with the highest prevalences of overweight and obesity (e.g. central and southern regions), as well as weather conditions (inland vs. seafront areas). The sample includes 18 interviews with the school staff, 11 interviews with parents, 6 focus groups with 58 parents (male = 7, female = 51), and 6 focus groups (4–8 participants per group) were conducted among 38 children (boys = 21, girls = 17) (Table 2). For the interviews and focus groups, a guide was developed based on the main study aims, which included an average of 24 items per instrument addressing the larger study aims, of which 9 were related to physical activity (Supplementary Material 1). Interviews with parents and teachers in Cuernavaca and head teachers in Toluca were conducted online, while the remaining interviews and focus groups were conducted in person.

**Table 2:** Number of schools, participants, focus groups, and interviews by city

Participants	Region						Total	
	Ciudad del Carmen		Toluca		Cuernavaca n (% female)			
	n Measurements	N (% female)	n Measurements	n (% female)	n Measurements	n (% female)	n Measurements	n participants
Schools		2		2		3		7
Focus groups with children	2	13 (46.2%)	2	11 (27.3%)	2	14 (57.2%)	6	38
Focus groups with parents	2	21 (100%)	2	12 (83.3%)	2	25 (80%)	6	58
Interviews with parents	3	3 (100%)	3	3 (66.6%)	4	4 (100%)	10	11
Interviews with generalist teachers	4	4 (50%)	5	5 (100%)	4	4 (75%)	12	13
Interviews with head teachers	2	6 (100%)	2	2 (50%)	2	2 (100%)	6	6

For the quantitative component, a questionnaire was designed to explore children’s physical activity frequency and preferences in and out of school, including items such as: *Outside of school hours*,* do you practice any physical or sports activities? (yes*,* no)*,* Outside of school hours*,* where do you usually practice that physical or sports activity? (home*,* street*,* sports center*,* park*,* local community center*,* public pitch*,* gym*,* cultural center)*.

### Data processing and analyses

For quantitative data, means and proportions were estimated. The interviews and focus group recordings were transcribed verbatim and analysed via inductive thematic analysis [29]. Two independent coders, one native Spanish speaker with professional English use (JG) and one native English speaker with professional Spanish use (FC), coded the data in English. After coding, discussions took place between the coders and the other three authors (GA, MB, and AJ), who acted as critical friends [30]. The first author, with experience in qualitative analysis, integrated the data and generated themes (GA). Illustrative text extracts were analysed in Spanish and translated into English (GA). The six phases of thematic analysis were taken into account [31].

### Trustworthiness

A critical friend’s procedure was used to ensure the scientific rigor of the qualitative analyses. This serves as an alternative to other techniques, such as interrater reliability or member-checking, owing to their limitations in terms of verification, trustworthiness, or reliability [32]. A critical friend’s role is to encourage reflection by asking critical questions to find alternative explanations and interpretations during data analyses [32]. In addition, triangulation of data sources between questionnaire responses and transcriptions was conducted to strengthen the data quality [33].

## Results

Both barriers and facilitators were integrated into three main themes: Theme (1) How children spend their time outside of school, Theme (2) Places that children access, and Theme (3) Social environment for physical activity outside of the school. The main identified barriers and facilitators vary according to the geographical region and informant (Table 3). The results of the questionnaire are summarised in Table 4.

**Table 3:** Shared and unique perceived barriers and facilitators for extracurricular physical activity in Cuernavaca, Ciudad Del Carmen and Toluca

Barriers							
	Shared	Unique			Per respondent		
		Ciudad del Carmen, Campeche	Cuernavaca, Morelos	Toluca, Mexico	Children	Parents	Teachers
	-Lack of time -Cost -COVID-19 lockdown-related changes leading to unavailability of opportunities. -Low parental support for enrolling or taking children to sports or recreational facilities. -Lack or limited availability of physical activity facilities. -Long distances to available physical activity facilities. -Lack of after-school programs or their suspension due to the COVID-19 lockdown. -Lack of social support	-Seasonal weather (extreme heat) -Low safety perception	-Low safety perception	-Seasonal weather (floods) -Poor road pavement conditions -Child labour and house chores -Long commutes	-Perception that their physical activity is mostly unstructured -Not having someone to play with -Adults’ lack of time to take them to parks or sports facilities. -Spare time spent on household chores	-Describe physical activity in relation to ongoing participation in structured physical activity -Lack of money to enrol their children to leisure or sport activities. -Lack of time to take their children to leisure or sport activities. -Lack of low cost or free programmes and facilities for physical activity and sports. -Low safety perception preventing them from spending time in parks or other outdoor facilities. -Children´s preference for screen-based recreational activities.	-Children spend their discretionary time working or supporting their parents with households, farming, or commerce. -Lack of extracurricular programs and those available were suspended due to COVID-19 lockdown. -Perception that parents cannot pay fees for sports clubs or other extracurricular physical activity. -Lack of adequate physical space and time to run school physical activity programmes. -It is not possible to run extracurricular activities at double-shift schools.
Facillitators							
	-Having social support -Active transportation	- Built environment support for active transport (e.g., cycling paths). -Teacher’s support. -Preference for sports. -Local physical activity programs. -Parents awareness of the importance of physical activity.	-Good weather. -Teachers encourage active transportation and other physical activities outside of school hours.	Good safety perception	Having friends or family to play with	-Physical activity and sports are perceived as important and good for health. -Intention to enrol their children to sports clubs	-Physical education teachers run sports tournaments -Generalist teachers leave active “homework”.

**Table 4:** Children questionnaire results

Engagement in sports and recreational physical activity outside of school hours		*N*(%)	Sex			Location			
		Total 36	Boys 17 17(47.2)	Girls 19 (52.8)		Campeche 19(52.8)	Toluca 9(25)	Cuernavaca 8(22.2)	
Participation									
	Children who participate	25 (69.4)	13(76.5)	12(63.2)		13(68.4)	6(66.7)	6(75)	
	Children whose schools offer extracurricular physical activities	0	0	0		0	0	0	
	Children who would like to have extracurricular sports organized by their school	6(16.7)	3(17.6)	3(15.8)		2(10.5)	3(33.3)	1(12.5)	
Active transportation									
	Children who walk to school	17(47.2)	8(47.1)	9(47.4)		11(57.1)	5(55.6)	1(12.5)	
	Children who ride to school	2(5.6)	1(5.9)	1(5.3)		0	2(22.2)	0	
Type of activity									
	Football	9(25)	6(35.3)	3(15.8)		4(21.1)	3(33.3)	2(25)	
	Basketball	2(5.6)	1(5.9)	1(5.3)		2(10.5)	0	0	
	Volleyball	0	0	0		0	0	0	
	Martial arts	1 (2.8)	1 (5.9)	0		1(5.3)	0	0	
	Gymnastics	1 (2.8)	0	1 (5.3)		1(5.3)	0	0	
	Dancing	2(5.6)	0	2(10.5)		1(5.3)	0	1(12.5)	
	Ballet	0	0	0		0	0	0	
	Swimming	1 (2.8)	0	1 (5.3)		1(5.3)	0	0	
	Skating	2(5.6)	1(5.9)	1(5.3)		0	1(11.1)	1(12.5)	
	Skateboarding	0	0	0		0	0	0	
	Running	4(11.1)	2(11.8)	2(10.5)		0	2(25.0)	2(22.2)	
	Riding a bike	3(8.3)	2(11.8)	1 (5.3)		1(5 3)	1(11.1)	1(12.5)	
	Other activities reported		Walking, walking the dog						
Places									
	Home	9(25)	5(29.4)	4(21.1)		3(15.8)	2(22.2)	4(44.4)	
	Street	4(11.1)	2(11.8)	2(10.5)		1(5.3)	1(11.1)	2(25.0)	
	Sports Centre	2(5.6)	1(5.9)	1(5.3)		1(5.3)	1(11.1)	0	
	Park	2(5.6)	2(11.8)	0		1(5.3)	0	1(12.5)	
	Public yards	2(5.6)	1(5.9)	1(5.3)		1(5.3)	0	1(12.5)	
	GYM	0	0	0		0	0	0	
	Community Centre	0	0	0		0	0	0	
	Other places reported	Academy, Church, Instructor´s house, relative´s house, beach, waterfront							
Frequency									
	One day per week	4(16)	2(15.4)	2(16.7)		3(23.1)	0	1(16.7)	
	Two days per week	1(4)	0	1(8.3)		0	1(16.7)	0	
	Three days per week	6(24)	4(30.8)	2(16.7)		2(15.4)	2(33.3)	2(33.3)	
	Four days per week	3(12)	1(7.7)	2(16.7)		2(15.4)	1(16.7)	0	
	Five days per week	9(36)	5(38.5)	4(33.3)		4(30.8)	2(33.3)	3(50)	
Reasons for engaging in physical activity^a^									
	Enjoyment	14(38.9)	7(41.2)	7(36.8)		8(22.2)	4(44.4)	2 (25.0)	
	Health	1(2.8)	1(5.9)	0		0	0	1(12.5)	
	Lose weight	1(2.8)	1(5.9)	0		0	0	1(2.8)	
	Other reasons reported	Self-defense, professional goals, homework from physical education teacher							
Reasons for not engaging in physical activity									
	Time	2 (5.6)	0	2(10.5)		2(10.5)	0	0	
	Homework	1 (2.8)	0	1(5.3)		1(5.3)	0	0	
	Safety	2 (5.6)	1(5.9)	2(5.3)		0	1(11.1)	1(12.5)	
	Economy	0	0	0		0	0	0	
	Lack of parks closely	3 (8.3)	2(11.8)	1 (5.3)		2(10.5)	1(11.1)	0	
	Lack of space at home	3 (8.3)	2(11.8)	1 (5.3)		2(10.5)	1(11.1)	0	
	Injury	1 (2.8)	1 (5.9)	0		1 (5.3)	0	0	
	Other reasons reported	Laziness, small house, the COVID-19 lockdown							
Home and neighbourhood facilitators									
	Children who consider home and neighbourhood easy for it	29(80.6)	13(76.5)	16(84.2)		14(79.3)	8(88.9)	7(88.5)	

Note.^a^= Other reasons were explored but yielded no responses and were removed from the table: competition, spending family time, spending time with friends, having a good time, and medical prescription

### Theme 1. How children spend their time outside of school

#### Child work-related activities

Outside the school, some of the children felt obligated to spend their discretionary time at their parents’ job or commerce. For example, support the family business, look after farm animals, maintain family goods, or perform house chores. Based on children’s expressions, it seems that they feel this is part of their routine and a sense of overall disagreement.

*“I simply have to accompany my parents to sell*,* nothing else” (Child*,* Cuernavaca*).

Teachers also perceived that some children are engaged in the family business, working in physically demanding and risky activities.

*“Some of them (children) help their parents*,* for example*,* in the afternoons at a store*,* right? Or well*,* mainly maybe those who make bricks*,* it’s like another type of activity that they’re doing as physical activity” (Teacher*,* Toluca).*

Other teachers indicated that children work in activities such as taking care of the family’s farm animals.

*“No one in my students attends any activity (extracurricular physical activity)*,* one of them takes care of the sheep*,* that is what they do…not all of them but are responsible for taking care of the sheep*,* whatever they need or taking them out” (Teacher*,* Toluca).*

This perception is shared by parents, who declare that their children engage in house chores such as mopping or cleaning as part of a routine after school.

*“from Monday to Friday they come back to their poor house and eat*,* and then they have to dedicate themselves to cleaning and taking out the little sheep*,* and then they play for a while*,* take a bath*,* do their homework*,* watch TV for a little while*,* -if they have time*,* otherwise*,* it’s time to sleep” (Parent*,* Toluca).*

Other parents indicate that these house chores are also part of children’s activities during school holidays.

*“My daughter*,* well*,* she cleans the little table where we eat*,* cleans the stove*,* I mean*,* she tidies up the whole kitchen in general (…)*,* And I just wash the dishes*,* and since my son is already on vacation*,* he helps me sweep*,* takes care of his dog*,* and sweeps his room and the patio” (Parent*,* Toluca).*

In addition to house chores, some parents indicated that their children engage in physically demanding activities, such as washing the family truck as part of the children’s routine mixed with screen time.

*“He (her son) helps his dad wash the truck every day*,* and then he spends some time on his phone” (Parent*,* Toluca).*

#### Lack of extracurricular programmes

Except for one school in Toluca that offered extracurricular volleyball, all the schools in the study did not offer extracurricular physical activity options, as reported by the head teachers. Among the main reasons are school double shifts or a lack of teachers. Head teachers also reported that open areas at school are crowded or have small courts. When head teachers refer to extracurricular physical activity, it is mostly by some parents’ initiative.

*“No*,* here at school it’s not feasible (having extracurricular activities). We used to leave at 2:30 pm*,* but now we leave at 12:30 pm*,* and then another school comes in and uses this facility from 12:30 pm onward” (Teacher*,* Morelos).*

#### Screens

Screen time was reported in the three states and across respondents. In terms of teachers’ perceptions, children spend a large amount of time outside of school alone at home, whereas parents work long or double shifts. Teachers believe that the time children spend alone at home is associated with screen-based forms of entertainment.

*“they stay at home*,* locked up*,* resorting to what they have*,* the television*,* the smartphone*,* the tablet (…)” (Teacher*,* Cuernavaca).*

This teacher perception is also shared with some parents reporting their children being alone at home engaged with screen-based forms of entertainment. Among the main uses are for social media or watching videos.

*“when I’m not at home*,* they can watch TV*,* and now that they have YouTube*,* they watch videos of children with toys” (Parent*,* Campeche).*

Parents also perceive that children spend a large amount of time at home on screens. Some parents reported that their child spent approximately 4 h per day watching TV. Other parents agreed that screen time with TV or smartphones was part of children’s daily routine. Some parents mention that their children are not physically active at home because their children would rather be in front of screens. In contrast, from the children’s perspective, they prefer being active, especially if they have someone to play with, whereas other children perceive their screen time as a reward.

*“Only on weekends*,* but the thing is*,* (…) in order to do that (using any screen)*,* I have to be constantly helping my mom during the week*,* and if not*,* then I can’t” (Child*,* Toluca).*

### Theme 2. Places that children access

#### House

A majority (80%) of the children reported in the questionnaire that it is easy to perform physical activity at their house and neighbourhood. The most common place where they perform physical activity outside of school is their home (25%), with a balance among girls (21.1%) and boys (29.4%). The second most common place reported was the street (11%). None of the participants reported access to community centres or gyms, whereas fewer than 6% went to parks or sports centres. Outside the school day, more children stay at home in Cuernavaca than in Ciudad del Carmen (44% and 15.8%, respectively). Children indicated that they commonly perform physical activity at their relatives, instructors, or own house, usually at patios or rooftops, common areas in housing complexes, streets, parks, empty lots, the beach, waterfront, churches, and private sports or art academies.

*“I take her (her neighbour) to my house*,* and we pretend we’re in dance classes” (Child*,* Campeche)*.

Among the main reasons reported in the questionnaire for not engaging in extracurricular activities were having a small house (8.3%), a lack of parks nearby (8.3%), and perceptions of neighbourhood safety (5.6%). Only children in Toluca and Cuernavaca reported concerns related to safety. Common areas within housing complexes were identified for engaging in physical activity. These common areas may offer facilities such as small parks, parking spaces, pavements, green areas, or open spaces. Residents of these private housing complexes usually pay a fee for the maintenance of common areas and security checkpoints.

*“What she does is go out to play*,* as it is a private housing complex where we live*,* she goes out to play here in the complex with her scooter” (Parent*,* Morelos).*

#### Outdoors

The waterfront was reported as important for families to spend their discretionary time being active outdoors in Campeche. Children reported engaging in activities such as walking, cycling, walking the dog, or enjoying open spaces. In contrast, parents in Morelos perceive that children do not have enough space to play at home.

*“Since I live in an apartment*,* they don’t have anywhere to run around” (Parent*,* Morelos).*

The use of open areas that children access varies by season and extreme weather. In Campeche, extreme heat prevented children from spending time outdoors before 6 pm, but cold weather prevented them from swimming. In Toluca, heavy rain prevented children from playing outdoors, riding, or walking for commuting.

*“(…) it is what they want or what the weather allows them*,* right now as it is raining a little earlier*,* they no longer go out” (Parent*,* Toluca).*

### Theme 3. Social environment for physical activity outside of the school

#### Family support

Parents’ support is essential for children to partake in unstructured physical activity outside school day *“(…) because it’s Sunday*,* I take them to the small park nearby where they play*,* run*,* jump*,* and relieve some stress”* (Parent, Morelos). Additionally, for structured activities, children reported how they rely on their parents to enrol or take them to the activities, and when this does not happen, children opt for screen-based activities or unstructured physical activities.

*“don’t do anything. Well*,* at the same time (…) because my mom didn’t get me into anything*,* but when I get home*,* I start to see the laptop*,* to see how to exercise or dance” (Child*,* Campeche).*

Some parents perceive that their time availability is a determinant of their ability to take their children to the park, sports, or other facilities for physical activity, particularly during weekdays. When parents are not available, they rely on relatives for this purpose. However, relatives’ work-related demands on weekends could also interfere.

***“(…)****right now as he (children´s uncle) no longer rest on Sundays*,* because he has to work*,* they have not gone to the park” (Parent*,* Toluca).*

Intangible support was identified as the opposite of expectations, where some parents discourage their children from engaging in physical activity, referring to them as lacking enough physical capability, weak, or too thin for certain activities. Tangible support is affected by economic limitations, as perceived by some parents, mainly to cover activity fees. However, in the questionnaires, none of the children reported that money was a reason to prevent them from taking part in physical activity outside the school.

*“To begin with*,* it would be the economy because we have to pay for them to be in such groups*,* because there are no free ones (extracurricular physical activities)” (Parent*,* Toluca).*

#### Parental modelling

Physically active parents showed positive attitudes toward enrolling their children in structured physical activity. Additionally, parents who are engaged in physical activity perceive fewer barriers regarding infrastructure, money, or time. This social environment seems to be positive for children’s engagement in physical activity outside the school day.

*“So*,* during my school years*,* I did a lot of sports*,* I participated in weightlifting*,* here at the Sports Institute. I worked out either before or after school. I participated in athletics*,* basketball*,* volleyball*,* weightlifting (…) I gave them (her children) options*,* and they loved the water. My children love to swim (…)” (Parent*,* Morelos).*

#### Active commuting

Approximately 5.6% of the participants reported riding a bike to commute to and from school. In the focus groups and interviews, riding a bike was mentioned to be common by teachers at Toluca only, and it was perceived as something special rather than routine, and something needed rather than a healthy option.

*“Well*,* yes a parade*,* they participated in decorating the bicycle…Then it was full of children and parents (…) I think they got borrowed bicycles” (Teacher*,* Toluca).*

According to the questionnaire data, 47.2% of the children walked to school. The reasons to walk to school as perceived by head teachers are living close to school. Similarly, riding a bike and walking to school is perceived as a need, as a parent mentioned in Campeche. However, in Toluca, walking to school is due to not having a bike and considering it expensive to purchase.

*“(…) to get here (the school) you must walk*,* there is no other option” (Parent*,* Campeche).*

Some teachers reported that many children arrive at school by car or foot. Children who arrive by car are perceived as having more favourable socioeconomic conditions or as those who live far from school.

*“(.) say that 80% already come by car*,* and it’s because our school is already made up of many students who come from outside and attend this school because their parents work there*,* so they take advantage of it*,* but the majority of the children are not from there” (Teacher*,* Toluca).*

## Discussion

This study aimed to explore the perceived barriers to and facilitators of physical activity outside of school among schoolchildren in Mexico. Three main themes summarise participants´ perceptions: Theme (1) How children spend their time outside of school, Theme (2) The places that children access, and Theme (3) Social environment for physical activity outside of the school. The perceived barriers and facilitators varied by informant and region. The main shared barriers were that children spend their spare time working, doing household chores, homework or engaging with screens; stay alone at home, lack access to school-based extracurricular physical activity programmes, live in small houses, lack physical activity facilities, limited time availability for both children and parents. The main shared facilitators were enjoyment of physical activity, having space to play at home, living in housing with open common areas, mild weather, living close to a beach, parental modelling, and family tangible and intangible support. Our findings also highlight that some children in Mexico have never attended any extracurricular programs outside of school. Additionally, a voice is given to children in Mexico who engage in economic responsibilities or risky household chores, which prevent them from engaging in healthier options such as active play, leisure physical activity or sports participation.

### Theme 1. How children spend their time outside of school

Overall, most children in the study generally spend their time outside the school, both during the school term and holidays, alone at home engaging in screen-based entertainment, unstructured physical activity, working with their parents, doing schoolwork or household chores, and accompanying their families at their commerce/business. The findings concerning children’s engagement in work-related activities outside of school are consistent with data from the National Survey Child Labour, which indicate that in Mexico, there are 25 million children and adolescents aged 5–17 years, 11.5% of whom engage in illegal or/and hazardous work [16]. The results from the 2019 edition of the survey also reported that child labour in Mexico is driven by poverty, discrimination, migration, cultural practices, a lack of appropriate jobs for adults, a limited number of schools, and inadequate social security [34]. Examples of the type of work conducted are agriculture, including farming and fishing, mining, construction, cleaning, and services. This 11.5% of child labour is above the average for the Americas (5%) and below that of the African region (19.6%) [35].These results resonate with the paradigm of necessity vs. choice-based physical activity [36], where although some, including children, may engage in physical activity, this is by necessity rather than by choice. A key feature of physical activity by necessity is that it is often performed under unsafe conditions, which make it difficult to enjoy. Additionally, it is often associated with a social class stigma towards those who engage in it. Indeed, recent studies in adults suggest that physical activity by necessity, especially occupational activity, could have adverse effects on health [37]. Occupational activity for adults may resemble child labour in terms of its compulsory nature, lack of choice, and potential for physical strain, leading to negative long-term health outcomes. In contrast, choice-based physical activity, such as sports, active play or structured physical activity, is considered more desirable for children in a health context, as it is typically associated with enjoyment, autonomy, and positive physical and mental benefits. Hence, this finding adds to the discussion raised by Valera and Halal, who question whether some forms of physical activity are health-enhancing and also congruent with dignity, cultural norms, and economic circumstances [38]. To improve children’s health and development and achieve the *sustainable development goal of ending child labour*, several social crises in Mexico must be addressed in the short term.

The findings of this study reveal that schools do not provide statutory extracurricular physical activity. Extracurricular physical activity refers to activities after the school day, during weekends, holidays, or school breaks [39]. Previous studies in HICs indicate that extracurricular physical activity could be led by teaching assistants (representing one-third of the teaching workforce in the United Kingdom) or sports coaches, who are usually funded by parents or schools [40]. Our study findings suggest that school staff consider extracurricular physical activity as a consideration for parents, but not necessarily the school’s responsibility. Denmark is the country with the highest documented participation of children and adolescents in organized sports worldwide. A key component of this success is the implementation of several national policies that support physical activity for children across various settings, including school, demonstrating the plausibility of this strategies for promoting active living among children [41]. The 2022 National Commission of Active Culture and Sports plan in Mexico is expected to launch School and Municipal Sports Centres to promote sports and physical activity among children, along with the School Physical Activations Programme, which could potentially address the lack of sport centers in the country [42]. In addition, only 51% of public elementary schools have a specialist physical education teacher [43]. Hence, allocating funds to reach national coverage of physical education teachers and integrate extracurricular physical activity nationwide for children’s health and well-being should be a top priority.

Another relevant finding in this study is screen usage as a form of entertainment outside the school day. A previous study among a sample of 3503 Spanish adolescents showed that as screen time increases, the time available for sports participation decreases [44]. Our study results suggest screen-based entertainment may replace extracurricular physical activity because they have greater access to screen-based devices and limited support for physical activity outside the school. Studies support the premise that limiting screen use limitations increases physical activity, as found among Danish children [45]. Our findings suggest that many children in Mexico spend time alone at home and unsupervised. In addition, although the study inclusion criterion included children from low socioeconomic backgrounds, having a smartphone was common. According to the 2020 National Survey on Availability and Use of Information Technologies in Households, 79.2% of Mexicans > 6 years of age use a smartphone [46]. This suggests that smartphones are an important item within Mexican families compared with washing machines, which are available in 71% of Mexican households [47]. The high availability of these technologies, regardless of family income, may be considered when designing m-health interventions; however, programmes to limit screen time should be a priority in the context of Mexico.

### Theme 2. Places that children access

This theme showed that across the three states, the home is the most common place for physical activity outside of school, whereas public parks or fee-based facilities are less popular. Locations on the front beach, mild weather, and living in a complex housing system were identified as important facilitators of physical activity outside of the school and outdoors. This preference for physical activity may be explained by other study findings, such as parents’ time constraints, the cost of structured activities, or low perceptions of neighbourhood safety. Another possible explanation is that risk aversion negatively impacts children’s opportunities for play and freedom as some cultures are more tolerant of risk than others [48], despite risky play being crucial for children’s healthy development [48]. Previous studies have shown that overprotective parenting styles negatively influence the risky play of their children [49]. Further studies are needed to explore the influence of Mexican parenting style and the engagement of children in active play outdoors or any other source of extracurricular physical activity.

With respect to the built environment, except for participants from Campeche, most children and their families were unfamiliar with free or low-cost nearby options for extracurricular physical activity in their neighbourhoods. Previous studies in a sample of preschool Latino children indicate that the proportion of MVPA is 43% greater when they are outdoors compared to indoors [50]. The availability of free or low-cost nearby options for extracurricular physical activity is low in Mexico. For every 100,000 inhabitants, there are only 10.4 public facilities dedicated to physical activity, physical culture, or sports [51]. Park availability ranges from 91 in Morelos to 209 in Campeche. Only 0.6% of the government administration budget is estimated to be allocated to sports and physical culture [52]. In Mexico, community facilities include institutes of sports, university programs, healthcare system programs, and physical culture and sports programs at the national level [53, 54]. Although these facilities are reported in official documents [55], there is limited information on the proportion of schoolchildren who access them nationwide.

### Theme 3. Social environment for physical activity outside of the school

Low parental tangible and intangible support for extracurricular physical activity and little or no physical activity culture at home were identified as social barriers. Active commuting is perceived as a necessity or as a special activity for those with better socioeconomic conditions. Parental tangible support for extracurricular physical activity was found to be fundamental to paying fees or taking children to the activities, and the main barriers perceived by parents were the cost and their time availability due to work duties. In terms of work, it is estimated that Mexicans work 2,207 h per year, whereas in other countries such as Germany, it is almost half of the hours (1,316) [56]. Mexico ranks last (41 out of 41) among OECD member countries in the working-life balance indicator [57]. Additionally, Mexico has the highest proportion of people working very long hours in paid employment, leaving them with less time for personal care and leisure compared to workers in other OECD member countries. Thus, increasing children’s physical activity demands structural changes in job-related laws that allow parents to have a work‒life balance.

Parental modelling was identified as essential for Mexican children’s participation in extracurricular physical activity. This finding was consistent with a previous study among 10,096 Spanish children aged 7–16 years, where having an active family member was associated with being enrolled in extracurricular sports [14]. Families’ voices in this study suggest that they engage in long working hours and weekend work, which may restrict their time availability to engage in extracurricular physical activity with their children. More research is needed on the direct effects of working hours and recreational physical activity among families in LMICs. The results of the present study suggest that families and school staff are unfamiliar with actively commuting for health and environmental purposes. Active commuting is instead perceived as an option for those living in disadvantaged circumstances, those who live nearby, or as part of special school events. Promoting active commutes to and from schools in Mexico may require addressing adults’ beliefs on this matter.

This is one of the first studies that provides a voice to children, their parents, and school staff about extracurricular physical activity in Mexico. It includes rich data on the perceptions of three different actors from three regions in Mexico. However, the results of this study should be interpreted with caution given several limitations. First, the cross-sectional study design limits our ability to explore processes or changes in participants’ experiences or perceptions over time since participants were asked to reflect on their experiences at a single point in time. Second, this study is part of a larger study with wider aims, limiting the possibility to explore in depth other aspects of physical activity, such as the influence of the characteristics of the built environment or physical activity programmes in the community to partake in physical activity outside the school day. Third, the schools were allocated by the Ministry of Education based on the criterion that these schools are usually responsive to this type of call. Finally, the view of those children living in rural areas may differ from the current findings.

## Conclusions

Many children in Mexico never partake in structured forms of physical activity outside of the school day. Instead, they often spend time on screen-based activities, work-related physical activity or unstructured physical activity, mainly at home. Children rely on their families to partake in extracurricular unstructured and structured physical activity. Given the effectiveness of extracurricular physical activity programmes in other contexts, integrating these initiatives as a national policy during school days and holidays is a priority in Mexico, not only to support schoolchildren´s health and well-being, but also to ensure their safety.

## Electronic supplementary material

Below is the link to the electronic supplementary material.

Supplementary Material 1

## Data Availability

The data that support the findings of this study are available upon request.
